# Overexpression of *miR17* ~ *92* in Myeloid Cells in Mice Increased Bone Mass Through Reduced Bone Resorption and Increased Bone Formation in Sex-Dependent Manner

**DOI:** 10.1007/s00223-024-01325-x

**Published:** 2025-01-03

**Authors:** Matilda H.-C. Sheng, Virginia M. Stiffel, Jordan Taipia, Charles H. Rundle, Kin-Hing William Lau

**Affiliations:** 1 Jerry L. Pettis Memorial VA Medical Center, VA Loma Linda Healthcare System, Loma Linda, CA USA; 2 Departments of Medicine and Biochemistry, Loma Linda University School of Medicine, Loma Linda, CA USA; 3 Musculoskeletal Disease Center (151), Jerry L. Pettis Memorial VA Medical Center, 11201 Benton Street, Loma Linda, CA 92357 USA

**Keywords:** Bone resorption, Sex specific, *miR17* ~ *92*, Osteoclasts, Overexpression

## Abstract

This study assessed the feasibility of *miR17* ~ *92*-based antiresorptive strategy by determining the effects of conditional transgenic (cTG) overexpression of *miR17* ~ *92* in myeloid cells on bone and osteoclasts. Osteoclasts of male and female cTG mutant mice each showed 3- to fivefold overexpression of *miR17* ~ *92* cluster genes compared to those of age- and sex-matched wildtype (WT) littermates. Male but not female cTG mutant mice had more trabecular and cortical bones as well as lower bone resorption reflected by reduction in osteoclast number and resorbing surface. Osteoclasts of male but not female cTG mutants showed decreased bone resorption activity. Consistent with suppression of osteoclast maturation, osteoclasts of male cTG mutants were smaller, contained less nuclei, showed reduced levels of mRNA of genes associated with osteoclast differentiation and fusion, and formed more diffused actin ring. Osteoclastic overexpression of *miR17* ~ *92* also increased bone formation, but the increase was much larger in males than in females. The increase in male mutants was due to higher mineral apposition rate, and conversely, it was caused by increasing bone-forming surface in female mutants. In summary, osteoclastic overexpression of *miR17* ~ *92* increased bone mass through reduction in bone resorption along with coupled increase in bone formation in male-specific manner. Although the osteoclastic overexpression of *miR17* ~ *92*-induced suppression of bone resorption and increases in bone formation support the feasibility of *miR17* ~ *92*-based antiresorptive strategies, the male-specific sexual disparity in skeletal responses to osteoclastic overexpression of *miR17* ~ *92* could limit its clinical utility as it may not be used in women with postmenopausal osteoporosis.

**Supplementary Information:**

The online version contains supplementary material available at 10.1007/s00223-024-01325-x.

## Introduction

Mammalian skeleton constantly undergoes regeneration to repair and replace fatigued or damaged bones through an important metabolic process known as the bone remodeling [1]. Bone remodeling is also needed for adaptation of the skeleton to changes in mechanical loading and is essential for the proper maintenance of structural and mechanical integrity of the skeleton. Bone remodeling is triggered by an initial increase in bone resorption followed by a compensatory increase in bone formation in a spatial and temporal manner. Information about the mechanism(s) regulating osteoclast resorption is essential for a better understanding of the overall regulation of bone remodeling.

Osteoclastic resorption is determined by the formation, survival, and activation of mature osteoclasts. Osteoclast formation and survival are regulated primarily by two local soluble factors released by osteoblasts and osteocytes, i.e., macrophage colony-stimulating factor (mCSF) and receptor activator of NFκB ligand (RANKL) [2–4]. Their respective signaling pathways are well documented [5–7]. Conversely, osteoclast activation is mediated by various cytokines in part through two intracellular pathways, i.e., the Src and the integrin β_3_ signaling. These two pathways work concertedly with each other [8, 9] and interact with other pathways, such as the immunoreceptor tyrosine-based activation motif and the immunoreceptor tyrosine-based inhibition motif pathways [8, 10], to modulate activation status of the osteoclast. The upregulation of Src signaling and the subsequent activation of integrin β_3_ signaling in osteoclasts is induced in part through the dephosphorylation of an inhibitory phosphotyrosine (pY) 527 residue (pY-527) of Src, which is catalyzed in part by an osteoclastic non-receptor-type transmembrane protein-tyrosine phosphatase [11–14] that is referred by us to as the PTP-oc [15]. PTP-oc can also act directly on EphA4 receptor to dephosphorylate its key pY residues, leading to an inactivation of its forward signaling [13], which is a potent negative regulatory mechanism of osteoclast activation [16]. Accordingly, the PTP-oc can further promote osteoclast activation through the suppression of the EphA4 forward signaling.

The PTP-oc in osteoclasts is regulated in part post-transcriptionally by *miR17*. Accordingly, the 3’-UTR of *PTP-oc* mRNA has a target site for *miR17*. The cellular level of *miR17* in osteoclasts was inversely correlated with that of *PTP-oc* mRNA [17]. Selective suppression of *miR17* mRNA with LNA antisense-based inhibitor in osteoclasts increased cellular PTP-oc level and bone resorption activity, whereas transgenic expression of pre-*miR17* ~ *92* in osteoclast precursors in vitro drastically reduced cellular *PTP-oc* level and decreased fusion and resorption activity of the derived osteoclasts [18]. The *miR17* is co-expressed as one of six cluster genes (i.e., *miR17*, *miR18*, *miR19a*, *miR19b*, *miR20*, and *miR92*) of the *miR17* ~ *92* cluster locus. Both male and female mutant mice with conditional knockout (cKO) of *miR17* ~ *92* genes in *Ctsk*-expressing mature osteoclasts showed lower Tb.BV/TV, Tb.BMD, Tb.Conn-Dens, Tb.N, larger Tb.Sp, and greater bone resorption without an apparent effect on bone formation compared to corresponding age- and sex-matched wildtype (WT) littermates [18]. The osteoclasts of *miR17* ~ *92* cKO mutants were twice as large, contained twice as many nuclei, and formed twice as large resorption pits as WT osteoclasts [18], suggesting that osteoclasts of these cKO mice were highly active. Therefore, *miR17* is a negative epigenetic regulator of *PTP-oc* and is an upstream mediator of the PTP-oc/EphA4 regulatory axis of osteoclast activation [19]. However, conditional disruption of the *miR17* ~ *92* cluster polycistronic genes in osteoclasts did not have negative impact on bone formation [18].

A key limitation of the current FDA-approved antiresorptive medications for osteoporosis is that these medications not only suppress bone resorption but also inhibit bone formation, limiting the amounts of new bone regeneration that replace the lost bone mass. Accordingly, these antiresorptive therapies offer only moderate regain of bone strength and reduction in fractures risks in the range of 20–40% [20, 21], which is lower than that accomplished by anabolic therapies, such as romosozumab (Evenity), which can be up to 75% [22]. Hence, an ideal antiresorptive therapy would be one that suppresses bone resorption, but by doing so would not negatively affect the coupled bone formation. That suppression of *miR17* ~ *92* (or *miR17*) expression upregulated bone resorption without inhibition of bone formation raises the interesting possibility that *miR17* ~ *92* (or *miR17*) could be an attractive target for such a novel antiresorptive therapy.

The present study sought to evaluate the feasibility of *miR17* ~ *92*-based antiresorptive strategy by determining the effects of conditional overexpression of *miR17* ~ *92* in cells of osteoclastic lineage on bone and osteoclast phenotypes. This work is necessary, because (1) the skeletal effects of other miRNA genes than *miR17* in the cluster have not been clearly defined, (2) the specificity and level of repression of a target mRNA achieved are dependent upon the amounts of both the target mRNA and the available miRNA complexes [23], (3) each miRNA targets multiple mRNAs, and 4) each target mRNA can be repressed by multiple miRNA species of the *miR17* ~ *92* polycistronic cluster. Thus, it is conceivable that high expression levels of *miR17* ~ *92* could lead to unexpected off-target effects and that overexpression of *miR17* ~ *92* in osteoclasts might not yield contrary effects on bone resorption as those of deletion of *miR17* ~ *92* in osteoclasts. Because osteoclasts are derived from cells of monocytic/macrophagic lineage of myeloid cells, we generated a colony of cTG mutant strain with conditional overexpression of *miR17* ~ *92* in lysozyme-M(LysM)-expressing myeloid cells by crossing *ROSA-miR17* ~ *92* mutant mice, which harbors a loxP-flanked neo-STOP cassette that prevents transcription of the downstream bicistronic human *miR17* ~ *92* cluster, with *LysM*-*Cre* transgenic mice for investigation. This study showed that *miR17* ~ *92* cTG mutant mice exhibited a high bone mass phenotype with reduced osteoclastic resorption and increased bone formation, but in a male-specific manner.

## Materials and Methods

### Materials

Culture media were purchased from Life Technologies (Grand Island, NY) and fetal bovine serum (FBS) was obtained from Hyclone Laboratories (Logan, UT) or Atlantic Biologicals (Flowery Branch, GA). Tissue culture supplies were from Falcon (Oxnard, CA). The enhanced chemiluminescence detection kit for Western blots was obtained from Millipore (Billevica, MA). Recombinant soluble receptor activator of NFκB ligand (RANKL) and macrophage colony-stimulating factor (mCSF) were from PeproTech (Cranbury, NJ). All other reagents were obtained from either Sigma-Aldrich (St. Louis, MO) or Thermo Fisher Scientific (Los Angeles, CA).

### Animals

Breeding pairs of *C57BL/6-Gt(ROSA)26Sor*^*tm3(CAG−MIR17−92,−EGFP)Rsky*^*/J* (*ROSA-miR17* ~ *92*, strain # 008517) was obtained from the Jackson Labs (Bar Harbor, ME). These mice harbor a loxP-flanked neo-STOP cassette preventing transcription of the downstream bicistronic human *miR17* ~ *92* cluster and enhanced GFP (*EGFP*) gene sequences that were inserted to the *Gt(ROSA)26Sor* locus, thereby allowing the Cre-inducible removal of the neo-STOP sequence to express the human *miR17* ~ *92* cluster genes. Conditional transgenic (cTG) mice overexpressing *miR17* ~ *92* cluster genes in myeloid cells were generated by breeding *ROSA-miR17* ~ *92* mice (in C57BL/6 genetic background) with *LysM-Cre* mice (in C57BL/6–129 genetic background), which were also purchased from the Jackson Labs (strain # 004781). The schematic representation of the breeding protocol is outlined in Suppl. Fig. S1. This protocol yielded 25% homozygous mutants (*LysM-Cre*^+^/*ROSA-miR17* ~ *92*^*flox/flox*^), 50% phenotypically normal WT littermates (*LysM-Cre*^−^/*ROSA-miR17* ~ *92*^*flox/flox*^ or *LysM-Cre*^*−*^/*ROSA-miR17* ~ *92*^*flox/−*^), and 25% heterozygotes (*LysM-Cre*^+^/*ROSA-miR17* ~ *92*^*flox/−*^). Homozygous mutants were used as the cTG mutants in this study. Phenotypically, normal WT littermates were used as controls for comparison. Mice are housed in 4 mice per cage in a 12-h light/12-h dark cycle and fed the regular Envigo/Harlan TekLad 7001 rodent diet (Placentia, CA, USA). All animal protocols (protocol # 0047/1160) were reviewed and approved by the Animal Care and Use Committee of VA Loma Linda Healthcare System. In conducting research using animals, the investigators adhered to the Animal Welfare Act Regulations and other federal status relating to animals and animal experimentation and the principles set forth in the current version of the National Institutes of Health (NIH) Guidelines for Research Involving Recombinant DNA Molecules. The funders had no role in study design, data collection and analysis, decision to publish, or preparation of the manuscript.

### Genotyping Assays

Tail vertebrate tissue (2–3 mm) was taken from each pup at weaning under anesthesia and was digested overnight using the DNeasy kit (Qiagen, San Diego, CA). The quality and quantity of genomic DNA were analyzed by the 260 nm/280-nm absorbance ratio. Genotyping of *ROSA-miR17* ~ *92* mutant mice was performed with the PCR-based assay recommended by the Jackson Labs using two primer sets [Wildtype—forward: CCA AAG TCG CTC TGA GTT GTT ATC; reverse: GAG CGG GAG AAA TGG ATA TG. Mutant—forward: ACC TCC CCC TGA ACC TGA AAC A; reverse: CAG TTT TAC AAG GTG ATG TTC TCT G]. Homozygote mutant mice showed a single band of 267 bp, heterozygote mutant mice showed two bands of 267 bp and 604 bp, respectively, and WT littermates showed a single band of 604 bp. *LysM*-*Cre* transgenic mice was genotyped according to the PCR-based assay recommended by the Jackson Labs using a set of three primers [common forward: CTT GGG CTG CCA GAA TTT CTC; WT reverse: TTA CAG TCG GCC AGG CTG AC; and mutant reverse: CCC AGA AAT GCC AGA TTA CG]. Homozygote mice showed a single band of 700 bp, heterozygote mutant mice showed two bands of 700 bp and 350 bp, respectively, and WT littermates showed a single band of 350 bp.

### Cell Cultures

Primary osteoclasts were generated from bone marrow osteoclast precursors of 10-week-old *miR17* ~ *92* cTG mutants or WT littermates according to the procedure described previously [16, 24]. The number and average size of tartrate-resistant acid phosphatase (TRAP)-positive, multinucleated (three or more nuclei), osteoclasts were measured for the entirety of each 24-well area at 4X magnification using the OsteoMeasure™ system (SciMeasure, Decatur, GA). TRAP activity of osteoclast-like cells were performed as previously described [24].

### Reverse Transcriptase-Quantitative Polymerase Chain Reaction (RT-qPCR)

The relative cellular mRNA levels of genes associated with osteoclastic differentiation, fusion, and activation were determined by RT-qPCR on BioRad CFX96 cycler using the SYBR Green method (Promega, Madison, WI) [13] and normalized against that of *β-actin*. The primer sequences of each test gene are shown in Suppl. Table S1. The data were analyzed with the comparative threshold cycle (ΔΔC_T_) method [25].

### Cellular miRNA Assays

The cellular *miR17 and miR19a* levels were determined as follows: TaqMan-based specific PCR primers with a stem-loop sequence for reverse transcription were purchased from Thermo Fisher Scientific (Los Angeles, CA, USA). Reactions containing 10 ng of total RNAs were incubated at 16 °C for 30 min., 42 °C for 30 min and 85 °C for 5 min. The qPCR using reverse-transcribed cDNA used as template was performed with a TaqMan Small RNA Assay kit (Thermo Fisher Scientific). Samples were incubated at 95 °C for 10 min, followed by 40 cycles of 95 °C for 15 s and 60 °C for 1 min. The relative level *miR17* or *miR19a* was normalized against *U6* RNA. Results are reported as fold of WT controls.

### Resorption pit formation Assay

The resorption pit formation assay was performed as described previously [26]. We focused on measurements of average pit area per pit (dividing total pit area by the number of pits), which represented the average bone resorption activity per osteoclast. The total pit area of 50–75 resorption pits in each dentine slice was determined using the OsteoMeasure™ system.

### Immunofluorescent Staining of Actin Rings of Osteoclasts

Marrow-derived osteoclasts of male cTG mutants or WT littermates, plated on uncoated glass slides, were paraformaldehyde-fixed and permeabilized with Triton X-100. The fixed cells were incubated with FITC-phalloidin at 37 °C in 1% DMSO in PBS. The immunofluorescent actin rings were visualized under a ZEISS Axio Observer fluorescent microscope.

### Micro-Computed Tomography (µ-CT) Measurements

Three-dimensional bone parameters were assessed on femurs with µ-CT using a Scanco vivaCT40 µ-CT scanner (Scanco Medical, Brüttisellen, Switzerland) as previously described [11]. Trabecular measurements were performed at the secondary spongiosa of distal femur (at a site that was 10% of the full length of the femur from the distal end). A region of 0.8 mm in thickness at 10% of the full length from the distal end was scanned. The trabecular masks were defined in a semiautomatic manner, starting from the outer mask of the femur and application of 15 erosion cycles to ensure that no cortex was included. Cortical measurements were performed at the midshaft of the femur.

### Bone Histomorphometry

Static osteoclastic (N.OC, N.OC/B.Pm, OC.Pm, and OC.Pm/B.Pm) and dynamic bone formation parameters (BFR, BFR/B.Pm, MAR, TLS, and MS/B.Pm) were measured at the secondary spongiosa of femur as previously described [24].

### Statistical Analysis

Results are shown as mean ± SEM. Statistical significance was determined with two-tailed Student’s *t* test or ANOVA followed by the Tukey post hoc test using the Systat 11 statistic software (Richmond, CA). Interaction and statistical significance between the effects of cTG and those Ca deficiency on µ-CT trabecular and cortical bone parameters were determined with two-way ANOVA. The difference was considered statistically significant when *p* < 0.05.

## Results

### *Overexpression of miR17* ~ *92 in LysM-Expressing Myeloid Cells Increased Bone Mass and Density in Male But Not Female Mutant Mice*

**Fig. 1:**
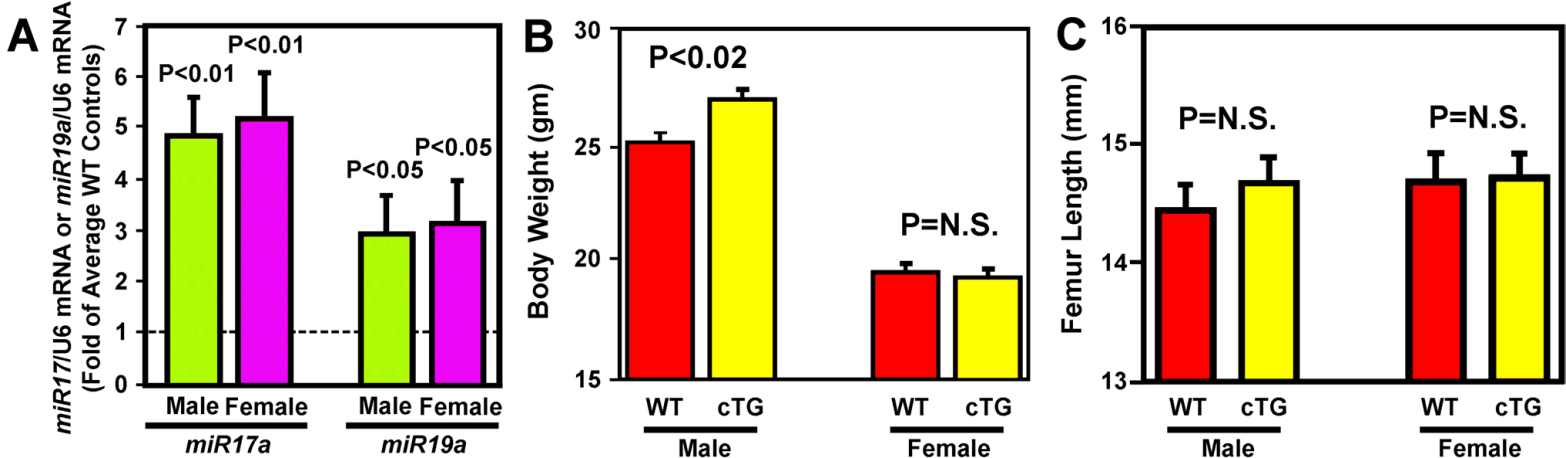
Cellular expression levels of *miR17* and *miR19a* in osteoclasts (**A**), the body weight (**B**), and femur length (**C**) of 10-week-old male and female *miR17* ~ *92* cTG mutants and corresponding WT littermates. A: Cellular levels of *miR17* and *miR19a* (normalized against U6 mRNA) in marrow-derived osteoclasts from 10-week-old male and female cTG mutants and WT littermates were measured as described in Methods. Results are shown as mean ± SEM (*n* = 5 for male cTG and *n* = 3 for female cTG mutants). The dashed line represents the level of each test miRNA of corresponding WT control cells. B: Results are shown as mean ± SEM (*n* = 14 for male WT, *n* = 7 for male cTG, *n* = 17 for female WT, and *n* = 5 for female cTG). C: Results are shown as mean ± SEM (*n* = 8 for male WT, *n* = 9 for male cTG, *n* = 5 for female WT, and *n* = 5 for female cTG). Statistical analyses were performed with two-tailed Student’s *t* test. P = N.S (statistically not significant, i.e., P > 0.05)

To determine the effects of conditional overexpression of *miR17* ~ *92* in osteoclasts on bone turnover and bone mass, a colony of transgenic (cTG) mutant mice with conditional *miR17* ~ *92* overexpression in myeloid cells was generated by breeding *ROSA-miR17* ~ *92* mice with *LysM-Cre* mice. The cellular levels of *miR17a* and *miR19a* in marrow-derived osteoclasts of male and female cTG mutants were ~ fivefold and ~ threefold, respectively, of those in osteoclasts of WT littermates of corresponding sex (Fig. 1A). The body weight of male, but not female, cTG mutants at 10 weeks of age was ~ 7% heavier than that of corresponding age- and sex-matched WT littermates (Fig. 1B). The femur length of both male and female cTG mice was not significantly different from that of WT littermates of corresponding sex (Fig. 1C). Thus, overexpression of *miR17* ~ *92* in osteoclastic cells may have a small enhancing effect on developmental growth in male mutants but did not negatively affect longitudinal bone growth of mutant mice.

**Fig. 2:**
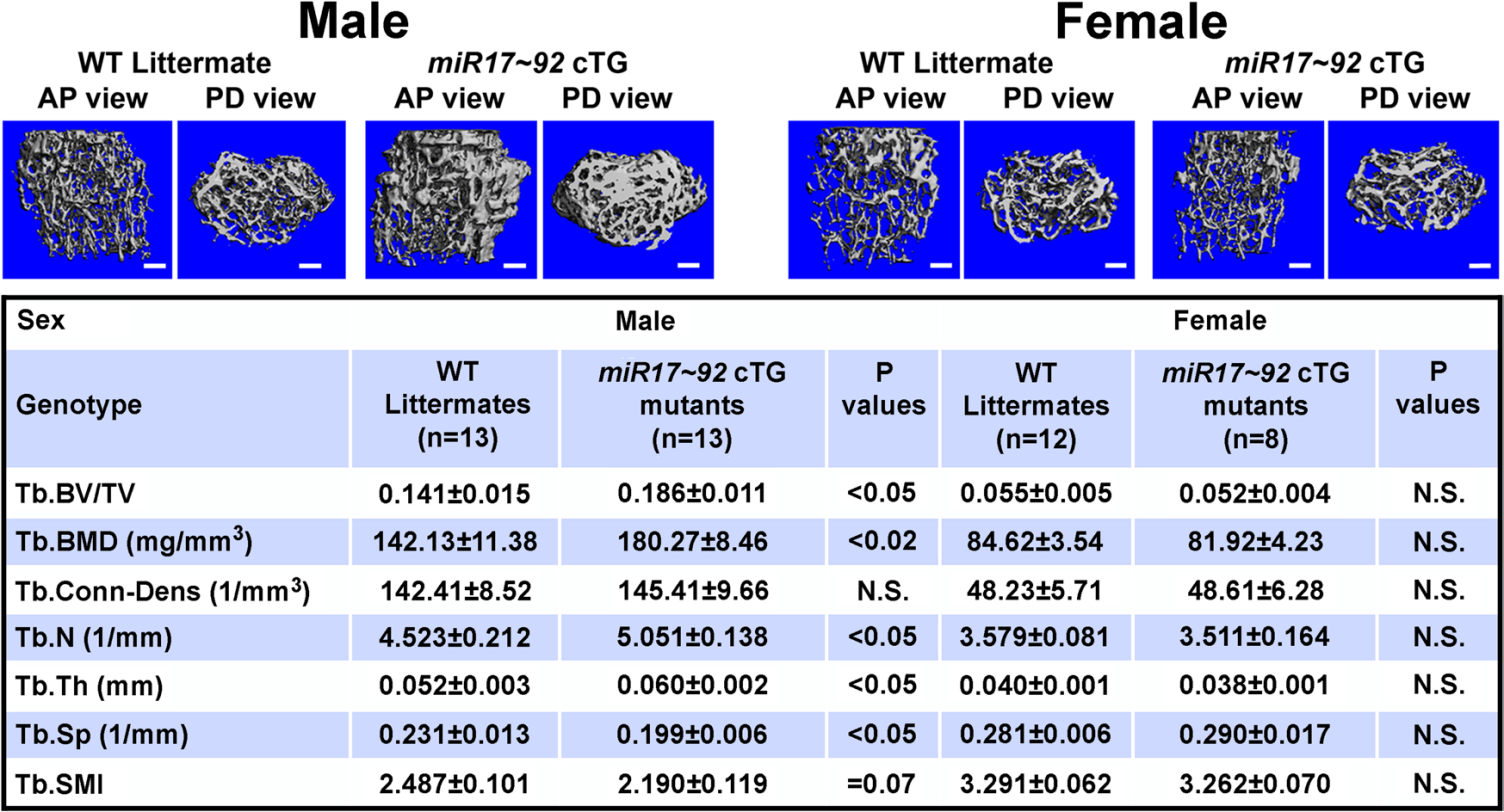
Three-dimensional µ-CT evidence that male and not female 10-week-old osteoclastic *miR17* ~ *92* cTG mutant mice had more trabecular bone than respective age- and sex-matched littermate control mice. Top pictures are anteroposterior (AP) and proximodistal (PD) views of the three-dimensional reconstruction of trabecular bone structure at the secondary spongiosa of distal femur of a representative male cTG mutant mouse, male littermate control mouse, female cTG mutant mouse and female WT littermate. Scale bar = 100 µm. Bottom tabulates the µ-CT trabecular bone parameters of male and female cTG mutant mice and corresponding WT littermates of respective sex. Tb.BV/TV = trabecular bone volume/tissue volume; Tb.BMD = trabecular bone mineral density; Tb.Conn-Dens = trabecular connectivity densities; Tb.Th = trabecular thickness; Tb.N = trabecular number; Tb.Sp = trabecular spacing; Tb.SMI = trabecular structural model index. Results are shown as mean ± SEM. Statistical significance was determined with two-tailed Student’s *t* test. P = N.S (statistically not significant, i.e., P > 0.05)

**Fig. 3:**
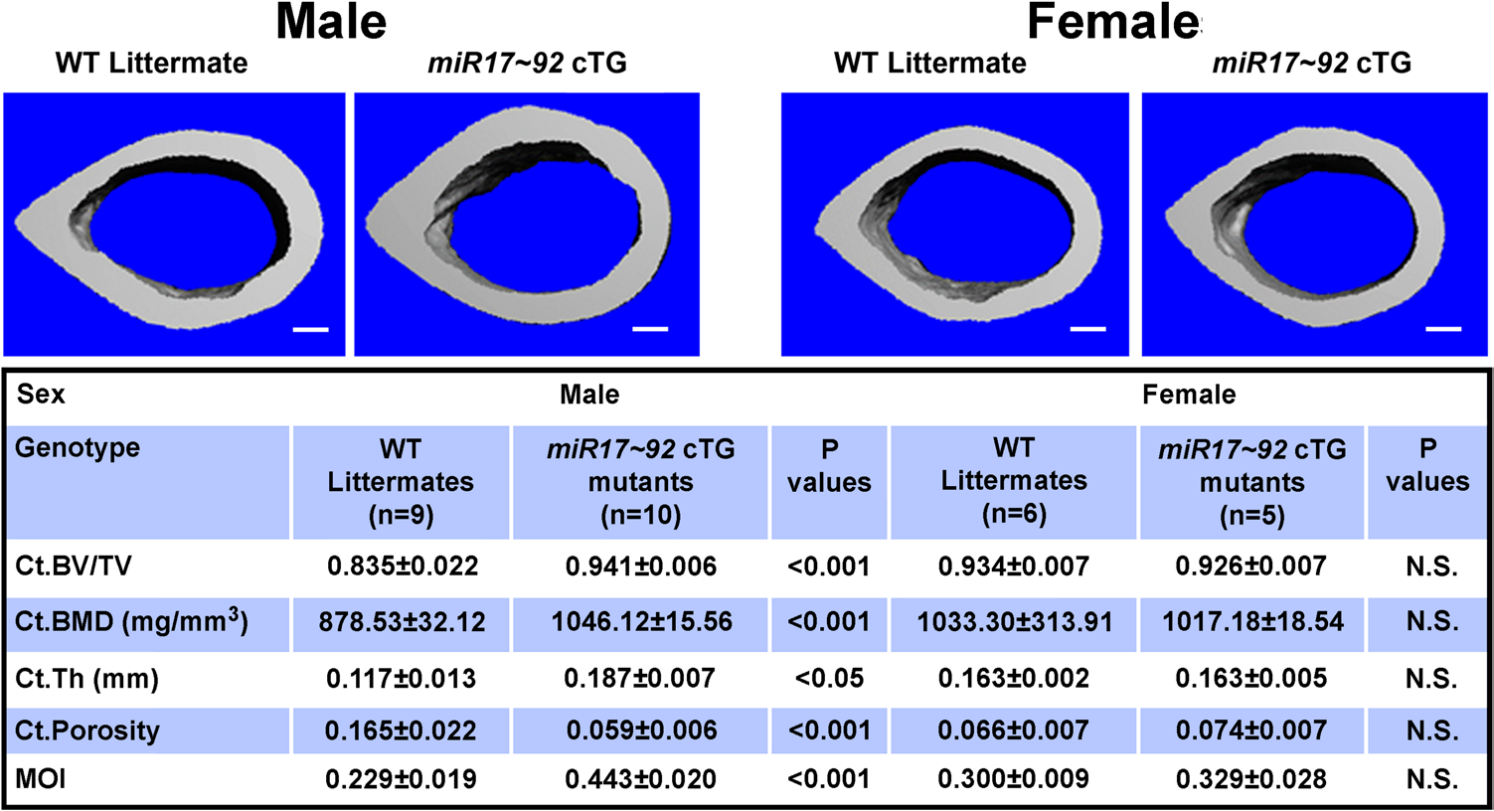
Three-dimensional µ-CT evidence that male but not female 10-week-old osteoclastic *miR17* ~ *92* cTG mutant mice had more cortical bone than respective age- and sex-matched littermate control mice. Top pictures are cross-sectional view of three-dimensional reconstruction of cortical bone structure at the midshaft of femur of a representative male cTG mutant mouse, male WT littermate, female cTG mutant mouse, and female WT littermate. Scale bar = 100 µm. Bottom tabulates the µ-CT cortical bone parameters of each group of mice. Ct.BV/TV = cortical bone volume/tissue volume; Ct.BMD = cortical bone mineral density; Ct.Th = cortical thickness; Ct.Porosity = cortical porosity; MOI = moment of insertia. Results are shown as mean ± SEM. Statistical significance was determined with two-tailed Student’s *t* test. P = N.S (statistically not significant, i.e., P > 0.05)

Figure 2 shows that trabecular bone at secondary spongiosa of distal femur of male cTG mutant mice showed greater Tb.BV/TV, higher Tb.BMD, thicker Tb.Th, more Tb.N, lower Tb.Sp, but similar Tb.Conn-Dens, compared to male WT littermates. Conversely, there were no significant differences in any trabecular bone parameters between female cTG mutants and female WT littermates. Figure 3 reveals that cortical bone at midshaft of the femur of male cTG mutants also had greater Ct.BV/TV, Ct.BMD, and Ct.Th, but lower Porosity than cortical bone of male WT littermates. In contrast, there were no significant differences in these cortical parameters between female WT littermates and female cTG mutants. Thus, the higher bone mass phenotype of cTG mutant was sex-specific and appeared to be restricted to male mutants only.

### Male But Not Female cTG Mutant Mice had Reduced Osteoclastic Resorption and Increased Bone Formation

**Fig. 4:**
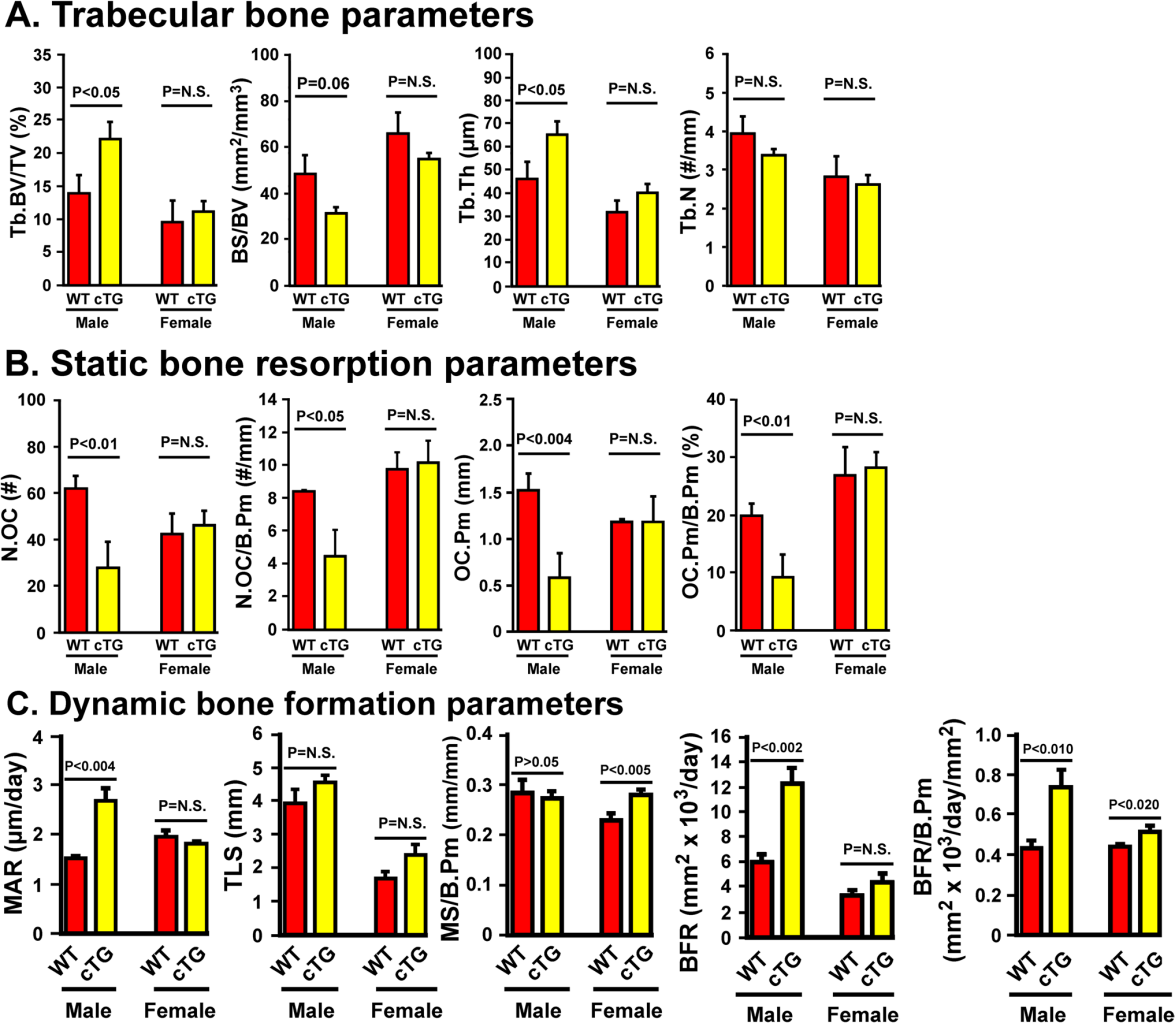
Bone histomorphometric parameters evidence that male and not female 10-week-old miR17 ~ 92 osteoclastic cTG mutant mice had increased trabecular bone volume and mass (**A**), reduced static osteoclastic parameters (**B**), and increased dynamic bone formation parameters (**C**) at the secondary spongiosa of distal femurs compared to respective age- and sex-matched littermate control mice. Results are shown as mean ± SEM. For bone volume and static bone resorption parameters, male cTG mutants: *n* = 5; male WT controls: *n* = 6; female cTG mutants: *n* = 7, and female WT controls: *n* = 7. For dynamic bone formation parameters, male cTG mutants: *n* = 6; male WT controls: *n* = 6; female cTG mutants: *n* = 6, and female WT controls: *n* = 6. Tb.BV/TV = trabecular bone volume per tissue volume; BS/BV = bone surface per bone volume; Tb.Th = trabecular thickness; Tb.N = trabecular number; N.OC = number of osteoclasts; N.OC/B.Pm = number of osteoclasts per bone surface; OC.Pm = osteoclast surface; OC.Pm/B.Pm = osteoclast surface per bone surface; MAR = mineral apposition rate; TLS = total labeling surface; MS/B.PM = mineralizing surface per bone surface (i.e., TLS/B.Pm); BFR = bone formation rate; and BFR/B.Pm = bone formation rate per bone surface. Results are shown as mean ± SEM. Statistical significance was determined with two-tailed Student’s *t* test. P = N.S (statistically not significant, i.e., P > 0.05)

Bone histomorphometry at the secondary spongiosa of distal femur confirms that male, but not female, cTG mutants exhibited increases in Tb.BV/TV and Tb.Th without an effect on Tb.N, and reduction in BS/BV (Fig. 4A). Static osteoclastic parameters, i.e., OC.Pm, OC.Pm/B.Pm, N.OC, and N.OC/B.Pm, were all reduced in male but not in female cTG mutants (Fig. 4B). Unless the cTG osteoclasts were overly active, the large reduction in OC.Pm/B.Pm and N.Oc/B.Pm would suggest that osteoclastic overexpression of *miR17* ~ *92* suppressed osteoclastic resorption. However, this was also limited only to male mutant mice. For dynamic bone formation parameters, male *miR17* ~ *92* cTG mutants increased BFR and BFR/B.Pm, which were caused largely by an increased MAR. Conversely, female *miR17* ~ *92* cTG mutants had a much smaller increase in BFR/B.Pm (Fig. 4C). Contrary to male mutants, the increase in BFR/B.Pm in female mutants was due to an increase in MS/B.Pm. In any event, the large increase in BFR/B.Pm, along with reduction in N.OC/B.Pm and OC.Pm/B.Pm, in male mutants led to a large and highly significant increase in Tb.BV/TV (Fig. 4A). In contrast, the small increase in BFR/B.Pm without significant changes in OC/B.Pm and OC.Pm/B.Pm in female mutants only resulted in a very small and statistically insignificant increase in Tb.BV/TV (Fig. 4A).

### *Overexpression of miR17* ~ *92 Suppressed Differentiation, Fusion, and resorption Activity of Osteoclasts*

**Fig. 5:**
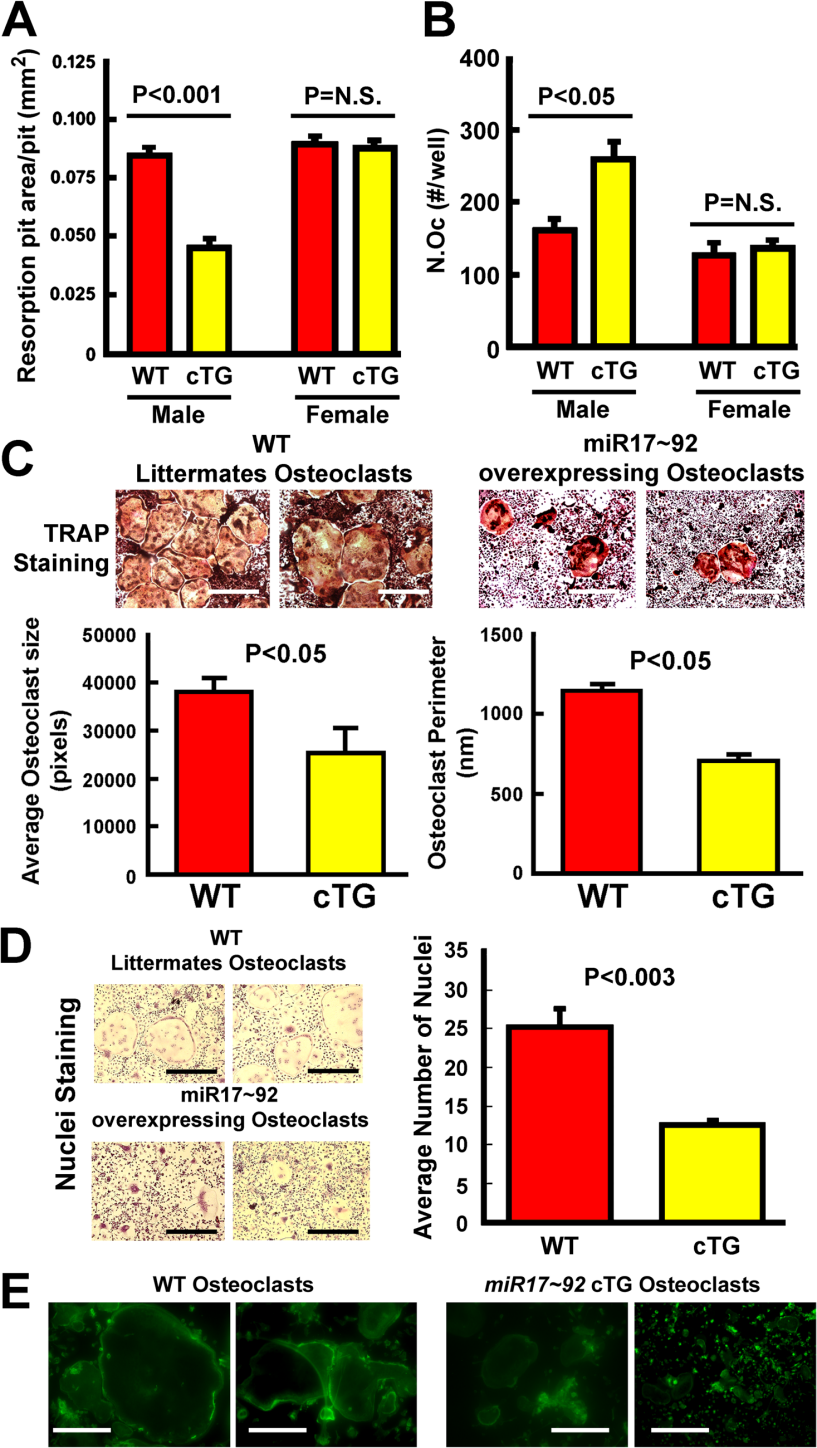
Marrow-derived osteoclast precursors of male but not female mutant mice with overexpression of *miR17* ~ *92* had reduced bone resorption activity (**A**), formed more (**B**) and larger (**C**) osteoclast-like cells with less average number of nuclei per cell (**D**) and more diffused actin ring (**D**) after the mCSF and RANKL treatment. In A, marrow-derived osteoclast precursors were plated on dentine slices and mCSF/RANKL were added sequentially to convert osteoclast precursors to osteoclasts. After one week of incubation, dentine slices were sonicated to remove attached cells and then stained with H&E. The resorption pit area per pit (as an index of osteoclast bone resorption activity) created by osteoclasts of *miR17* ~ *92* cTG mutant mice and WT littermates of both male (*n* = 7 per group) and female (*n* = 4 per group) was measured with the OsteoMeasure™ system. In B-E, osteoclast precursors were plated on 24-well plates and treated with mCSF/RANKL for 5–7 days. In B-D, the number, area, and perimeter and number of nuclei (after toluidine blue staining) of osteoclasts formed were measured with the OsteoMeasure™ system. Top panel of C shows photomicrograph of TRAP-stained osteoclasts of representative cultures of WT littermates and *miR17* ~ *92* cKO mutants. Scale bars = 20 µm. Panel D is toluidine blue-stained osteoclasts of representative cultures of WT littermates and *miR17* ~ *92* cKO mutants. Scale bars = 20 µm. In E, actin ring of osteoclasts was identified by immunofluorescent staining. Scale bars = 10 µm. Panels D to E were done with osteoclasts derived only from male cTG mutants or male WT littermates because osteoclasts of female cTG mice did not show significant osteoclastic phenotype as shown in panels A&B. Results in A to D are shown as mean ± SEM. P values were determined with two-tailed Student’s *t* test. P = N.S (statistically not significant, i.e., P > 0.05)

The average resorption pit area per pit created by marrow-derived osteoclasts of male cTG mutants, and not those of female cTG mutants, was significantly smaller than that of osteoclasts derived from WT littermates of respective sex (Fig. 5A), indicating that overexpression of *miR17* ~ *92* reduced the in vitro bone resorption activity of osteoclasts but also in male-specific fashion. Despite reduced resorption activity, the mCSF/RANKL treatment of same number of osteoclast precursors of male cTG mutants yielded more (Fig. 5B), but smaller (Fig. 5C) TRAP-positive, osteoclast-like cells with less average number of nuclei per cell (Fig. 5D) than those of WT littermates, suggesting that *miR17* ~ *92* overexpression suppressed fusion or spreading of osteoclasts that led to the reduced bone resorption activity. Consistent with the interpretation of reduced osteoclastic activity, the cTG osteoclasts formed more diffused actin rings (Fig. 5E). In contrast, there were no differences in the average size, average number of nuclei per cell, or actin ring formation between osteoclasts derived from female cTG mutants and those of female WT littermates (data not shown).

**Fig. 6:**
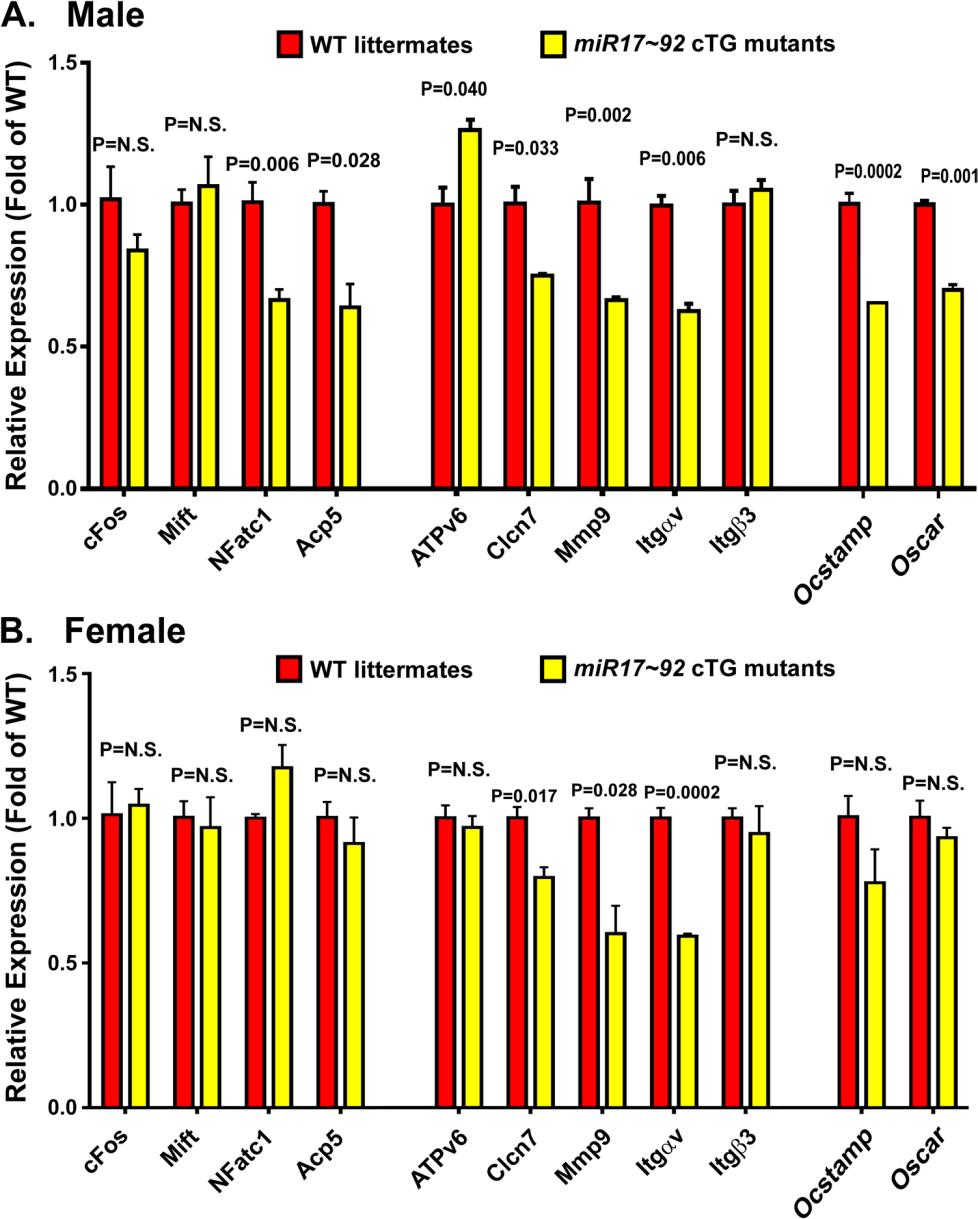
Comparison of relative expression levels of osteoclastic genes associated with differentiation, activation, or fusion in marrow-derived osteoclasts of male (**A**) and female (**B**) *miR17* ~ *92* cTG mice with those in osteoclasts of age- and sex-matched WT littermates. The relative expression level of each test osteoclastic mRNA was determined by RT-qPCR of total RNA extracted from marrow-derived osteoclasts of *miR17* ~ *92* cTG mice, normalized against respective β-actin mRNA. Results are shown as relative fold of the expression level of corresponding mRNA in osteoclasts of WT littermates (mean ± SEM, and *n* = 4 mice per group). A: Gene expression levels in osteoclasts of male cTG and WT littermates. B: Gene expression levels in osteoclasts of female cTG and WT littermates. Statistical significance was determined with two-tailed Student’s *t* test. P = N.S (statistically not significant, i.e., P > 0.05)

To evaluate if overexpression of *miR17* ~ *92* in osteoclasts suppresses their differentiation, fusion, and activity, the relative mRNA levels of several genes known to be associated with osteoclastic differentiation, fusion, and activity, respectively, were measured by RT-qPCR. Figure 6A shows that the mRNA levels of *Clcn7*, *Mmp9*, and *Itgav* [genes associated with osteoclast activation] in male cTG osteoclasts were significantly lower than those in male WT osteoclasts. The mRNA level of the other two osteoclast activity genes (*ATP6v0d2 and Itgb3*) was either slightly increased or no difference in male cTG osteoclasts compared to those in male WT osteoclasts. The mRNA levels of *Nfatc1* and *Trap* (*Acp5*), but not *Fos* and *Mitf* [genes associated with osteoclastic differentiation], were also reduced in male cTG osteoclasts compared to those in male WT osteoclasts. The mRNA level of two osteoclast fusion genes (i.e., *Ocstamp and Oscar*) in male cTG osteoclasts was similarly lower than those in male WT osteoclasts. Conversely, overexpression of *miR17* ~ *92* in osteoclasts in female mutants did not significantly affect the mRNA levels of genes associated with osteoclastic differentiation or fusion (Fig. 6B), which is consistent with the lack of significant differences in the N.Oc formed from the same number of marrow-derived precursors in response to the mCSF/RANKL between female cTG mutants and female WT littermates (Fig. 5B). However, the deletion of *miR17* ~ *92* in osteoclasts of female cTG mutants significantly reduced mRNA level of some genes associated with osteoclastic activity, i.e., *Clcn7*, *Mmp9*, and *Itgav*, compared to osteoclasts of corresponding female WT mice (Fig. 6B), but without significantly altered the resorption pit formation activity of the female cTG mutant osteoclasts (Fig. 5A). The reason for these apparent conflicting observations is not clear. It might be that the relatively small reduction in the mRNA level of these highly abundant osteoclastic genes was insufficient to affect the overall cellular protein levels of these functional genes to mediate the bone resorption activity.

**Fig. 7:**
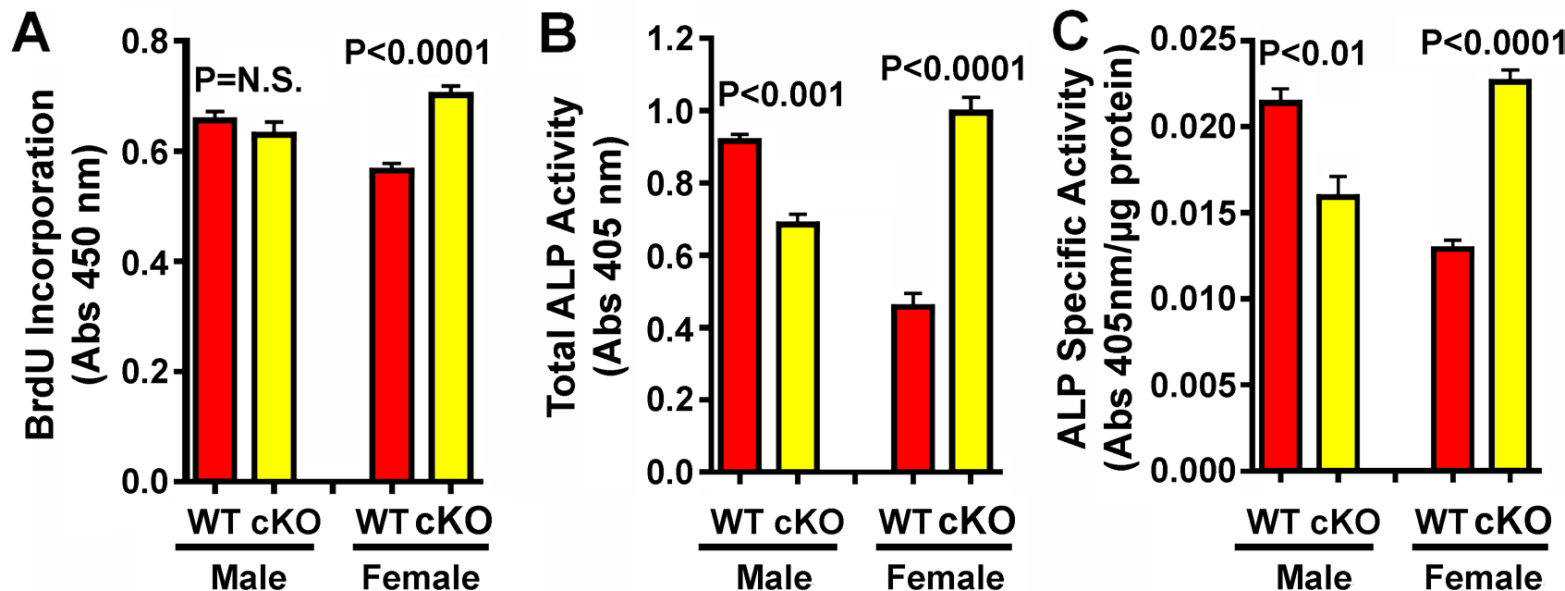
Effects of overexpression of osteoclastic *miR17* ~ *92* on basal proliferation and differentiation of primary osteoblasts isolated from 9-week-old male and female cTG mutants and corresponding WT littermates in vitro. A: The proliferation of primary osteoblasts was assayed with a BrdU Incorporation ELISA assay kit during the final 17 h of the 24 h of incubation. B&C Cellular alkaline phosphatase (ALP) activity of primary osteoblasts of male and female cTG and WT littermates after 48 h of incubation using an enzymatic activity assay as described in [24]. B shows total cellular ALP activity per well and C represents cellular ALP activity per µg cellular protein. Results are shown as mean ± SEM (n = 6 per group). Statistical significance was determined by two-tailed Student’s *t* test. P = N.S (statistically not significant, i.e., P > 0.05)

*Overexpression of miR17* ~ *92 in Myeloid Cells Affected the Proliferation and Differentiation of Osteoblasts Derived from cTG Mutants also in Sex-Specific fashion* Fig. 4C shows that *miR17* ~ *92* overexpression in osteoclastic cells caused sex-specific increase in bone formation. To determine if osteoclastic *miR17* ~ *92* overexpression would indirectly alter the proliferation and differentiation of primary osteoblasts isolated from cTG mutant mice, the BrdU incorporation (a measure of cell proliferation) and cellular alkaline phosphatase (ALP) activity (an accepted osteoblastic differentiation marker) of primary osteoprogenitors isolated from male and female *miR17* ~ *92* cTG mutants and WT littermates of corresponding sex were determined. Figure 7A shows that BrdU incorporation into osteoblasts of female but not osteoblasts of male cTG mutants was increased compared to those of WT littermates of corresponding sex, indicating that osteoclastic overexpression of *miR17* ~ *92* indirectly altered the proliferation of osteoblasts, also in a sex-specific manner that was seen only in female mutant mice. Similarly, both total cellular ALP activity per well (Fig. 7B) or cellular ALP-specific activity (Fig. 7C) of female cTG mutants were significantly greater than those of female WT littermates. Conversely, both total cellular ALP activity and cellular ALP-specific activity of male cTG mutants were significantly reduced compared to those of male WT littermates. Increased proliferation and osteoblastic differentiation would increase the number of functional osteoblasts on bone surface. The increases in BrdU incorporation and ALP activity of osteoblasts of female but not male cTG mutants are consistent with the increases in TLS and MS/B.Pm in female but not male cTG mutants (Fig. 4C).

## Discussion

This study clearly demonstrated that osteoclastic overexpression of *miR17* ~ *92* led to large increases in bone mass in both trabecular and cortical bones and caused > 50% reduction in histomorphometric osteoclast parameters in male cTG mutant mice. This study also showed that osteoclasts derived from male mutants were smaller, contained less nuclei per cells, more diffused actin rings, and reduced their in vitro bone resorption activity than osteoclasts derived from corresponding male WT littermates. Accordingly, while large, multinucleated, TRAP positive but entirely inactive osteoclasts could be found on bone surface under certain pathological and genetic mutation situations, the findings of large reductions in N.OC/B.Pm and OC.Pm/B.Pm in male cTG mutant mice, along with substantial decreases in the in vitro resorption activity of their marrow-derived osteoclasts, support our premise that osteoclastic overexpression of *miR17* ~ *92* suppresses osteoclast maturation and their bone resorption activity. Moreover, male cTG mutant mice exhibited substantial increases in bone formation. The fact that osteoclastic overexpression of *miR17* ~ *92* not only did not negatively impact bone formation but in fact it promoted bone formation raises the exciting possibility that local administration of *miR17* ~ *92*-based drugs could potentially not only exert antiresorptive activity but also osteogenic actions. Consequently, *miR17* ~ *92* (or *miR17*) mimics could be developed into an attractive, novel antiresorptive therapy that also possesses osteogenic actions.

This study, however, also made two unexpected discoveries that may cast some doubts about its overall clinical utility. First and foremost, the high bone mass/density and low osteoclastic resorption phenotype in these cTG mutant mice was sex-specific that was seen only in males. This sexual disparity in the skeletal response was unexpected because our previous study [18] indicates that cKO mutants with targeted deletion of *miR17* ~ *92* in osteoclasts showed no sex-specific differences in bone or osteoclast phenotype. However, there have been reports of sexual dimorphism in mice with targeted deletion of *miR17* ~ *92* in proopiomelanocortin-expressing neurons in the hypothalamus in the phenotype of aggravation of fatty diet-induced obesity only in male mutants and yielded sex-related differential gene expression profiles in responsive to high-fat diet between male and female neurons [27]. Conversely, the expression of the *miR17* ~ *92* cluster genes in the liver of C57BL/6 mice also showed sexual disparity in that much higher expression was seen in female than male mice [28]. Because osteoclasts derived from male or female cTG mutants did not show significant differences in the cellular expression levels of *miR17a* and *miR19a*, the cause for the sex-specific differential skeletal response between male and female cTG mutant mice was probably not due to a sexual disparity in the expression levels of *miR17* ~ *92* cluster genes in osteoclasts. It has also been reported that the sex-specific upregulation of *miR17* ~ *92* cluster genes expression in the liver was estrogen-responsive and mediated through the ERα signaling [29]. Accordingly, it is conceivable that sex hormones, especially estrogen, may have a conferring role in the sexual disparity in the skeletal response to osteoclastic overexpression of *miR17* ~ *92*, albeit the exact nature of the interaction between *miR17* ~ *92* overexpression and the signaling of sex hormones remains undefined. It should be noted that osteoclasts derived from *miR17* ~ *92* cTG mutants also displayed similar male-specific reduction in in vitro bone resorption activity. This may suggest that the male-specific response to *miR17* ~ *92* overexpression could be intrinsic to the osteoclast. If it is true, one may argue against a key role for systemic factors, including sex hormones. Regardless of the mechanism, the fact that the antiresorptive action of *miR17* ~ *92* overexpression was seen only in males indicates that the *miR17* ~ *92*-based strategy may not be effective or suitable for use in female patients with postmenopausal osteoporosis. However, we should be mindful that the 10-week-old adolescent mice used in this study had functional ovaries that produced abundant levels of estrogen, which may have played a suppressive role in the skeletal response to *miR17* ~ *92* overexpression in female mutant mice. It is entirely conceivable that under situations when circulating levels of estrogen are depleted as in the case for postmenopausal women or ovariectomized animals, the effects of the *miR17* ~ *92* overexpression are unleashed and therefore yielded the anticipated skeletal effects. Accordingly, additional studies with estrogen-depleted (i.e., ovariectomized) animals would be needed before a more definitive conclusion can be made on whether the *miR17* ~ *92*-based strategy can be an effective antiresorptive therapy for postmenopausal osteoporosis.

Secondly, overexpression of *miR17* ~ *92* in osteoclasts not only did not suppress but greatly enhanced bone formation, suggesting that the *miR17* ~ *92* overexpression-induced suppression of bone resorption did not appear to have inhibitory effects on the coupled bone formation, but the mechanism by which osteoclastic overexpression of *miR17* ~ *92* acted to promote bone formation is not clear. However, previous studies have shown that *miR17* ~ *92* has key regulatory roles in bone formation, albeit the effects appeared conflicting. On the one hand, conditional disruption of *miR17* ~ *92* in type-I collagen-expressing osteoblastic cells reduced periosteal bone formation and loading-induced osteogenic response [30]. On the other hand, antagonizing *miR17* ~ *92* with an *anti-miR17-92* oligo-augmented PTH-induced bone gain and restored bone mass in a mouse model of osteoporosis [31], implicating an inhibitory role of *miR17* ~ *92* in PTH-mediated bone formation. In any event, the fact that overexpression of miR17 ~ 92 in osteoclastic cells caused an increase in bone formation in cTG mutant mice suggests that *miR17* ~ *92* released by osteoclasts could act indirect and distally on osteoblasts to promote bone formation. Mechanistically, it has been well established that miRNAs can be secreted by living cells in the form of exosomes to migrate to and be taken up by neighboring or distal cells to affect their cellular functional activities [32, 33]. It is conceivable that overexpression of *miR17* ~ *92* in osteoclasts could increase their releases of *miR17* ~ *92*-containing exosomes to impact on neighboring or distal osteoblasts to promote bone formation. This intriguing possibility has merits and will be investigated in our future studies.

A salient fact of the foregoing observation is that the increased bone formation response to osteoclastic overexpression of *miR17* ~ *92* also displayed sexual dimorphism, since the increase in bone formation was much larger in male than in female mutant mice. Mechanistic cause for the sexual disparity on bone formation is unclear, but it may also be related to potential interactions between osteoclast-derived *miR17* ~ *92* and sex hormones. However, despite osteoclastic overexpression of *miR17* ~ *92* yielded much smaller increase in bone formation in female cTG mutants compared to male mutants, isolated osteoblasts from female mutants showed greater increase in BrdU incorporation and ALP-specific activity than those of male cTG mutants, suggesting greater basal osteoblast proliferation and differentiation in female cTG mutants. The mechanistic reason for this apparent discrepancy is not apparent, but it does suggest that the sexual disparity on bone formation in these cTG mutant mice was not due to intrinsic changes in osteoblastic proliferation and differentiation in response to osteoclastic overexpression of *miR17* ~ *92*. Moreover, there appeared to be distinctive sex-specific differences in potential mechanism(s) for the enhanced bone formation in these cTG mutants. Accordingly, the increased bone formation in male cTG mutants was caused primarily by an increased MAR, whereas that in female mutants was associated largely with an increased TLS (or MS/B.Pm). MAR is an index of osteoblastic activity, and TLS (or MS/B.Pm) is an index of bone-forming surface, which represents an increase in the number of active osteoblasts on bone surface. The finding that osteoblasts of female but not male cTG mutants increased the proliferation and differentiation of osteoprogenitor cells is consistent with the increase in TLS and MS/B.Pm in female but not male cTG mutant. Regardless of the mechanism, the reduced effects of osteoclastic overexpression of *miR17* ~ *92* on bone formation in female mutants further indicates that *miR17* ~ *92*-based strategy is probably less effective in and not suitable for female patients with postmenopausal osteoporosis.

This study has several caveats with respect to the intrinsic issues with the experimental design. First, the *miR17* ~ *92* polycistronic cluster simultaneously expresses 6 different miRNAs. Each of these 6 miRNAs has specialized functions in conferring cellular functions in normal development and in various diseases [34, 35]. While the regulatory role(s) of each of these 6 miRNAs in bone metabolism have not been clearly established, there is evidence that these miRNAs have regulatory functions in both bone formation and resorption. For instance, *miR17* in osteoclasts plays a potent inhibitory role in osteoclast activation [18, 19]. In osteoblasts and mesenchymal stromal cells, *miR17* suppresses osteogenic differentiation of osteoprogenitors by targeting Smad5 and increases bone formation in ovariectomized mice [36]. *MiR17* in osteoblastic cells also inhibited BMP-induced heterotopic ossification [37, 38]. The *miR18* in stromal cells inhibited osteoblastic differentiation and bone formation [39], whereas *miR19a* and *miR19b* in osteoblastic cells have positive regulatory role in bone formation [40] and *miR20* promoted osteogenic differentiation and bone formation [41]. Consequently, this study did not allow definitive conclusions as to whether the observed increased bone formation phenotype and/or sex-dependent changes in the bone mass phenotype was caused by *miR17* or by any single one or combination of more of the other 5 miRNAs.

Secondly, each of the 6 miRNAs targets multiple mRNAs, and each target mRNA can be repressed by multiple miRNAs depending on the relative expression of the miRNAs and target mRNA. These 6 miRNAs can also interact with each other to affect various cellular activities and functions [34, 35]. The expression levels of *miR17a* and *miR19a* in osteoclasts of both male and female cTG mutants were rather high (i.e., 3 to 5 folds). Because the expression of all 6 cluster genes of the *miR17* ~ *92* cluster was believed to be controlled by the same promoter, it is reasonable to assume that the expression of the other 4 genes of this cluster would also be highly elevated. Accordingly, we cannot rule out the possibility that some or all the foregoing effects (including sexual disparity) could be artifactual due to high expression levels of these other miRNAs of the *miR17* ~ *92* polycistronic locus.

In conclusion, this study demonstrated for the first time that conditional overexpression of *miR17* ~ *92* in myeloid cells led to substantial gain of both trabecular and cortical bone mass and density through suppression of bone resorption along with an increase in bone formation. While these findings support the feasibility of *miR17* ~ *92*-based antiresorptive strategies, the sexual disparity in the skeletal response that was seen only in males cast doubts on its potential clinical utility of the *miR17* ~ *92* overexpression-based antiresorptive therapy for bone-wasting disorders, especially for women. However, the current study evaluated the combined effects of simultaneous overexpression of all 6 polycistronic genes of the *miR17* ~ *92* locus. The expression levels of these miRNAs in cTG mutants were also highly elevated. Therefore, we cannot rule out the possibility that moderate increase in the cellular level of *miR17* alone, e.g., the use of *miR17* mimics, could avoid the foregoing undesirable effects, including the sexual disparity in the skeletal response. Consequently, our future study will explore the feasibility of *miR17*-based antiresorptive strategies for osteoporosis and related bone-wasting diseases and determine whether increased local bone concentrations of *miR17* alone would suppress bone resorption and increase bone formation but without sexual disparity.

## Supplementary Information

Below is the link to the electronic supplementary material.

Supplementary file1 (DOCX 68 KB)

## Data Availability

The research data that support the findings of this study is stored at an approved storage facility within the VA Loma Linda Healthcare system and are available from other investigators for review upon reasonable request and approval by the VA Loma Linda Healthcare system.
