# Supplementary material for: Overexpression of miR17 ~ 92 in Myeloid Cells in Mice Increased Bone Mass Through Reduced Bone Resorption and Increased Bone Formation in Sex-Dependent Manner
Source: Calcif Tissue Int. 2025 Jan 3;116(1):9. doi: 10.1007/s00223-024-01325-x (PMC11698891; doi:10.1007/s00223-024-01325-x)
Supplement: Supplementary file 1 — Supplementary file1 (DOCX 68 KB) [file 223_2024_1325_MOESM1_ESM.docx]

**Supplemental Information**

**to**

**Overexpression of *miR17~92* in Myeloid Cells in Mice Increased Bone Mass Through Reduced Bone Resorption and Increased Bone Formation in Sex-Dependent Manner**

Matilda H.-C. Sheng,^1,2^ Virginia M. Stiffel,^1^ Jordan Taipia,^1^ Charles H. Rundle,^1,2^

and Kin-Hing William Lau.^1,2^

^1^Jerry L. Pettis Memorial VA Medical Center, VA Loma Linda Healthcare System, Loma Linda, California, U.S.A; and

^2^Departments of Medicine and Biochemistry, Loma Linda University School of Medicine, Loma Linda, California, U.S.A.





Supplemental Figure S1. The schematic illustration of the breeding strategy of osteoclastic *miR17~92* conditional overexpression transgenic mice.

**Supplemental Table S1. Sequence of PCR primer sets for the test mouse genes.**

| Gene | Gene Accession # | Forward Primer | Reverse Primer | Tm (^o^C) |
| --- | --- | --- | --- | --- |
| *PTP-oc* | U32587.1 | 5'-TGACTTTAGCCGAGTGAGATTAC-3' | 5'-GGTGAGTTGTACCCAGGAATATAG-3' | 60 |
| *Cre* | DQ023272.1 | 5’-GTGTAGAGAAGGCATTAGC-3’ | 5’-CTGACCAGAGTCATCCTTAG-3’ | 60 |
| *Atp6v0d2* | NM_175406.3 | 5'-TGGACATGACCACAACCTGCAGTA-3' | 5'-TCGCACAGAGGGATCCATGAAGTT-3' | 60 |
| *Calcr* | NM_007588.2 | 5'-TACTTCCCGGACTTTGACACAGCA-3' | 5'-TCGGTTGCTGTCAGGGTGTCTAAA-3' | 60 |
| *Clcn7* | NP_036060 | 5’-ATCGTGGCCTTCATAGAGCCTGTT-3’ | 5’-ATCCTGAGTGGATCATTGGCCCTT-3’ | 60 |
| *Itgb3* | EDL34227 | 5’-TTCAATGCCACCTGCCTCAACAAC-3’ | 5’-ACGCACCTTGGCCTCGATACTAAA-3’ | 60 |
| *Mmp3* | AAO37583.1 | 5’-AGCTGAGGACTTTCCAGGTGTTGA-3’ | 5’-ACACAGGATGCCTTCCTTGGATCT-3’ | 60 |
| *Oscar* | AAI37777.1 | 5’-GTCAGGCTTGTTGAAGGATTAAAG-3’ | 5’-AAGGCACAGGAAGGAAATAGAG-3’ | 60 |
| *Oc-Stamp* | AB109560 | 5’-TGGGCCTCCATATGACCTCGAGTAG-3’ | 5’-TCAAAGGCTTGTAAATTGGAGGAGT-3’ | 60 |
| *Rank* | NM_009399.3 | 5’-TAGGACGTCAGGCCAAAGGACAAA-3' | 5’-AGGGCCTACTGCCTAAGTGTGTTT-3' | 60 |
| *Fos* | NM_010234 | 5’-TTGATGGATCCGGCCTTGCAAATG-3’ | 5’-TATGTTGGGAAGGTTGGCTGGACA-3’ | 60 |
| *Nfact1* | AF239169.1 | 5’-AGATGGTGCTGTCTGGCCATAACT-3’ | 5’-TGGTTGCGGAAAGGTGGTATCTCA-3’ | 60 |
| *Acp5* | BC029644.1 | 5’-AAGAACTTGCGACCATTGTTAGC-3’ | 5’-AGCCGCCCAGGGAGTCCTCAGATC-3’ | 60 |
| *Mitf* | NM_001113198.1 | 5’-ACATGTCCACTGGAAGCCCAGTAA-3’ | 5’-ATGAACGTATGAGGCCAGTGAGCA-3’ | 60 |
| *TNFα* | D84199.2 | 5’-TAGGACGTCAGGCCAAAGGACAAA-3’ | 5’-AGGGCCTACTGCCTAAGTGTGTTT-3’ | 60 |
| *Traf6* | D84655.1 | 5’-AGCTGTCCTCTGGCAAATATC-3’ | 5’-GTTGGGCAGTCCAGATCATAA-3’ | 60 |
| *Ppia* | NM_008907 | 5’-AATAGAAGCAGCAGGACCTGGGAA-3’ | 5’-AAAGCAAAGCCTGCCAGAAGACAC-3’ | 60 |
